# Classification of oil palm fresh fruit maturity based on carotene content from Raman spectra

**DOI:** 10.1038/s41598-021-97857-5

**Published:** 2021-09-15

**Authors:** Thinal Raj, Fazida Hanim Hashim, Aqilah Baseri Huddin, Aini Hussain, Mohd Faisal Ibrahim, Peer Mohamed Abdul

**Affiliations:** 1 Department of Electrical, Electronic and Systems Engineering, Faculty of Engineering and Built Environment, Universiti Kebangsaan Malaysia, 43600 Bangi Selangor, Malaysia; 2 Department of Chemical and Process Engineering, Faculty of Engineering and Built Environment, Universiti Kebangsaan Malaysia, 43600 Bangi Selangor, Malaysia

**Keywords:** Electrical and electronic engineering, Raman spectroscopy

## Abstract

The oil yield, measured in oil extraction rate per hectare in the palm oil industry, is directly affected by the ripening levels of the oil palm fresh fruit bunches at the point of harvesting. A rapid, non-invasive and reliable method in assessing the maturity level of oil palm harvests will enable harvesting at an optimum time to increase oil yield. This study shows the potential of using Raman spectroscopy to assess the ripeness level of oil palm fruitlets. By characterizing the carotene components as useful ripeness features, an automated ripeness classification model has been created using machine learning. A total of 46 oil palm fruit spectra consisting of 3 ripeness categories; under ripe, ripe, and over ripe, were analyzed in this work. The extracted features were tested with 19 classification techniques to classify the oil palm fruits into the three ripeness categories. The Raman peak averaging at 1515 cm^−1^ is shown to be a significant molecular fingerprint for carotene levels, which can serve as a ripeness indicator in oil palm fruits. Further signal analysis on the Raman peak reveals 4 significant sub bands found to be lycopene (ν1a), β-carotene (ν1b), lutein (ν1c) and neoxanthin (ν1d) which originate from the C=C stretching vibration of carotenoid molecules found in the peel of the oil palm fruit. The fine KNN classifier is found to provide the highest overall accuracy of 100%. The classifier employs 6 features: peak intensities of bands ν1a to ν1d and peak positions of bands ν1c and ν1d as predictors. In conclusion, the Raman spectroscopy method has the potential to provide an accurate and effective way in determining the ripeness of oil palm fresh fruits.

## Introduction

The oil extracted from the oil palm (*Elaeis guineensis*) contributes to approximately 1/3 of the world’s vegetable oil, followed by oil extracted from soybean and rapeseed^1,2^. Most of the extracted oil comes from the oil palm mesocarp, followed by oil extracted from the kernel^1^. Palm oil has significant importance in world trade, with usage not only limited to the food industry, but also to cosmetics, pharmaceutical products, etc.^2,3^. Despite an average extraction level of only 25%, palm oil still produces the world’s highest yield of oil per unit area, compared to the rest of the leading oil-bearing crops^3,4^.

To increase the production of good quality crude palm oil, one of the challenges is to harvest the oil palm fresh fruit bunches at the optimal ripened stage^5,6^. Currently, the method used to determine the optimal ripened stage is by color and loose fruits observation. If the fruit mesocarp outer most layer turn to a yellowish orange color and if around 10 loose fruitlets have detached from their sockets and fallen on the ground, it means the fruit bunch is ready to be harvested^7^. This conventional method is highly dependent on the unchartered technique of the palm fruit grader’s experience and intuition to determine the ripeness accurately which is not easily repeatable and prone to significant human error. In addition, color observation is only feasible for shorter trees and is highly dependent on sufficient illumination. Moreover, the observed detached loose fruits could have been washed away from another tree by heavy rain, eaten by animals or stuck in between fronds^8^.

Various studies have been made in the past to find a systematic solution to determine oil palm fruit ripeness that is cost-efficient, rapid, non-invasive, reliable and precise. Earlier methods in detecting the maturity of oil palm fruits can be broadly classified into two categories: non-optical and optical. The non-optical methods are either based on capacitive sensing^9^, inductive sensing^10–13^ and resistive sensing^14–16^. Non-optical methods usually measure the basic molecular content of all fruits such as moisture, which influences the overall performance of the system. The optical detection method can be further subdivided into 3 groups: imaging^5,17–21,21–26^, spectroscopy^6,27–30^ and spectral imaging^31–35^. Among all the methods introduced earlier, imaging is the most popular method to date due to its portability, low cost, and ease of implementation. However, the quality of the image is subjected to different lighting environments such as sun light exposure, illumination and shadow. Next to imaging, spectroscopy has gained increasing attention among researchers, such as absorption spectroscopy method using a portable ultraviolet–visible (UV–Vis) spectrometer^6^ and NIR spectroscopy^29^. Besides absorption property, measurement of the fluorescence property of the oil palm bunch using fluorescence sensor has also been previously introduced^30^.

Recently, researchers have demonstrated the use of Raman spectroscopy in assessing the ripeness and freshness of tomatoes and citruses by detecting the molecular vibrations of carotenoids and other chemical compounds found in the skin of the fruits^36–38^. These studies were demonstrated using both confocal Raman spectrometer and handheld Raman spectrometer. A Raman-based device uses high intensity light to measure the inelastic scattering from the surface of a targeted compound, which is later used as its molecular fingerprint. Most recently, a real-time monitoring for early detection of nutrients deficiency was demonstrated via a leaf-clip based Raman probe^39,40^. All studies have shown that the molecular vibrations can be associated with the Raman shifts that represent key organic compounds such as carotenoids, chlorophyll, nitrogen, etc. which were concluded based on the raw Raman spectra.

However, a separate study in biological chemistry has shown that each of this raw Raman spectrum are made up of convoluted signals, contributed by different organic compounds^41^. For example, β-carotene, lycopene, lutein and neoxanthin all could be seen as one convoluted peak on the highest Raman spectrum at around 1515 cm^−1^. Deconvoluting this spectrum and further synthesizing the signals could lead to a more accurate classification of key organic compounds.

In this study, a confocal Raman spectroscopy instrument is used to analyze the organic characteristic in the exocarp of oil palm fruitlets. In our previous study^42^, by analyzing the raw Raman spectra, we have concluded that carotene content is one of the major indicators that contribute to determining the ripeness level of oil palm fruits. Here, we demonstrate that the carotene peak shown by the Raman spectrum could be further deconvoluted and processed to identify four carotenoid components, which are β-carotene, lycopene, lutein and neoxanthin. These components and their derivations are extracted as features for our classification model based on machine learning, to automatically identify the ripeness level of the oil palm fruits.

## Methods

### Oil palm fruit/preparation

The oil palm fruit samples were collected from an oil palm plantation owned by the National University of Malaysia (UKM) and managed by its commercial arm, Khazanah UKM. The samples taken are from the *Elaeis guineensis* DxP species, which is a hybrid between the *Elaeis guineensis fo. dura* (thick-shelled) and the *Elaeis guineensis fo. pisifera* (shell-less) species. The Malaysian Palm Oil Board (MPOB) grading standard was used as a guideline for identifying the ripeness levels for under ripe, ripe, and over ripe fruitlets^7^. The under ripe fruit was chiseled from the bunch since it is hard and intact. The ripe fruits were collected from a harvested bunch while the over ripe fruits were collected from detached fruitlets. All fruitlets were inspected and verified by a Khazanah UKM in-house grader. A total of 46 samples were collected with a combination of 14 under ripe, 20 ripe and 12 over ripe samples. Upon collection and verification of the fresh fruitlets, the samples packed and tagged to be transferred to the laboratory for sample preparation. Due to the lack of fiber optic probe, the samples were not scanned in in-situ. Instead, the oil palm fruit samples were prepared into a thin layer for Raman scanning. This was accomplished by peeling a thin layer of the skin (exocarp) from the oil palm fruitlet using a scalpel, which was then transferred to a microscope slide so that it can be placed under the Raman microscope.

### Raman instrumentation

The Raman spectra were collected using a benchtop confocal Raman spectrometer (Thermo Scientific, DXR Raman Microscope). The spectrometer was set up using a 532 nm laser, green filter, 900 lines/mm grating and 50 µm slit aperture. The samples were exposed for 3 ms for 3 times, with laser power of 2.0 mW. For each sample, the spectra were collected from 3 distinct regions namely top, middle and bottom of the fruitlet.

### Raman spectral processing

The raw spectra obtained from the Raman instrumentation were then treated using signal processing methods (pre-treatment) to remove noise. First, the spectra received baseline corrections using rubber band algorithm. This step is necessary to remove any zero offsets present in the spectra. Next, the baseline-corrected spectra were treated using a smoothing process. This is achieved using the second order of Savitzky–Golay filter with filter size of 9 points. This process ensures the elimination of high frequency noise present in the spectra. Since the essential features are embedded in the C=C stretching vibration band, the entire spectra were segmented to 1495 to 1535 cm^−1^ Raman shift range. Next, the segmented spectra went through a deconvolution process using curve fitting techniques. For this process, a sum of 4 Lorentzian profiles were used. The profiles were placed manually and intuitively to serve as the initial parameters for curve fitting. The deconvoluted spectra contain variations in peak positions, which were then calibrated using the peak position of the pure β-carotene^43^ as a reference marker.

### Statistical analysis

Statistical analysis was applied to investigate the significance of each feature towards assessing the ripeness of the fruitlets. The statistical analysis employed in this study consists of homogeneity test, one-way ANOVA and post hoc test. Homogeneity test was used to detect the difference in variance among the 3 ripeness categories. If such a difference is detected, a further test using Brown-Forsythe and Welch were performed to validate the results. Next, one-way ANOVA was conducted to analyze the means of the selected features. This analysis helps to filter out features having the least contribution towards determining the ripeness of oil palm fruit. Finally, a multi comparison analysis was conducted as a post hoc test to identify features that contribute significantly to the ripeness differentiation. Based on previous research, the least significant difference test was preferred for samples that have equal sizes^30^. However, we employ the Gabriel test because our sample size is non-uniform. Further Games–Howell test was also performed to test the robustness of the features when unequal variance was detected. The statistical significance of features computed in statistical analysis was used to develop feature selection policy for the classification analysis.

### Classification analysis

Classification analysis for predicting the ripeness level was performed using an exploratory approach as shown in Fig. 4 in the Appendix. In the first phase of classification analysis, the extracted Raman features were selected and divided into test and training data using a fivefold validation scheme. The default policy for selecting features from full Raman features dataset was initialized to full statistical significance. A total of 19 classifier algorithms were initialized for training and validation in the second phase. The classifiers were taken from classification and regression trees (CRT), support vector machines (SVM), K-nearest neighbor classifiers (KNN), ensemble classifiers and discriminant analysis (DA). For each classifiers the performance score was computed and stored. In the final phase, the scores of classifiers were evaluated, and top 3 classifiers were filtered based on the maximum score. The process was repeated with a policy update for selecting new features until the target score was met. The target score was set to 100% prediction accuracy.

## Results

### Oil palm fruit spectra

**Figure 1:**
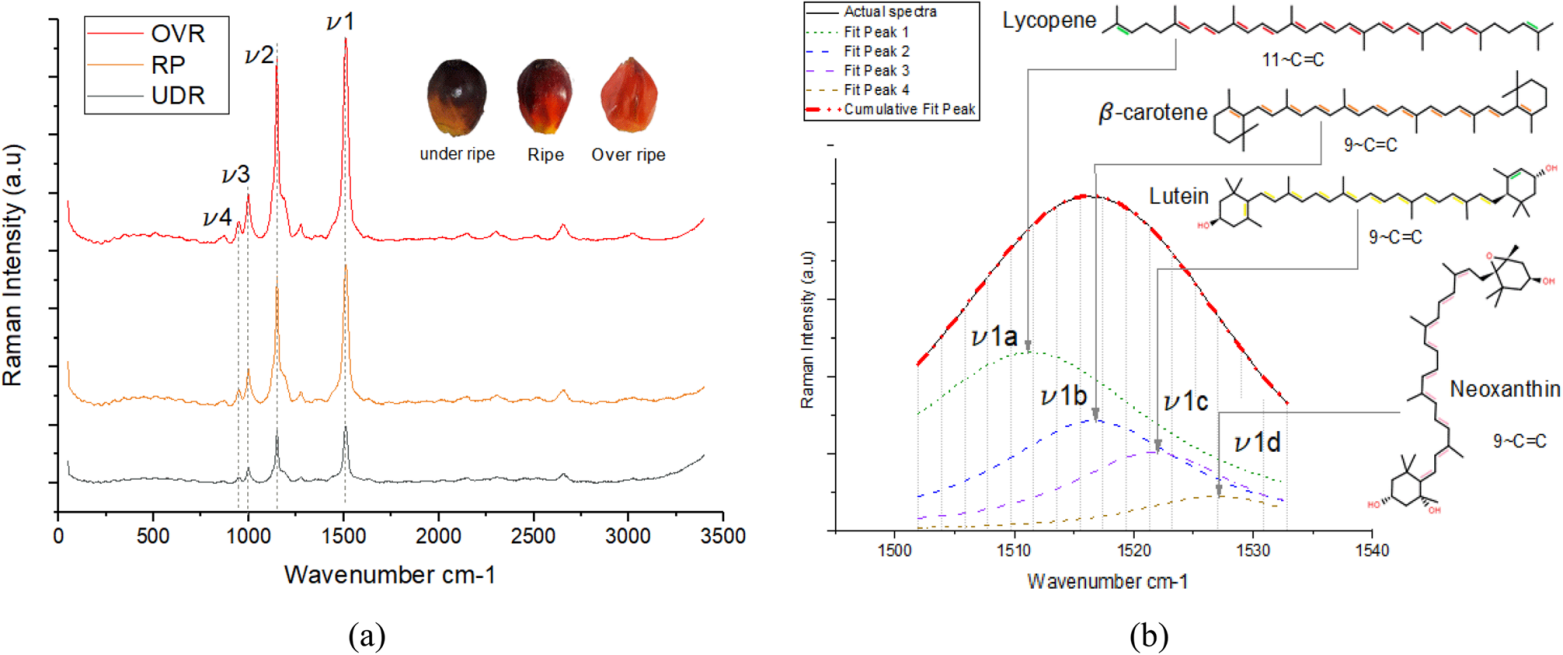
(**a**) Oil palm fruit Raman spectra of 3 levels of ripeness (*OVR* over ripe, *RP* ripe, *UDR* under ripe). (**b**) Deconvolution results for C=C stretching vibration band and its sub band molecular assignments.

The overall spectra obtained from the 3 levels of ripeness are shown in Fig. 1a. For all the 3 levels of ripeness, four intense vibrations were observed at locations averaging at 958, 1000, 1150 and 1515 cm^−1^. These vibrations are consistent with the characteristics of carotenoid molecules^41,44–46^. The strongest peak, ν1, located at 1515 cm^−1^ originates from the C=C stretching vibration of the carotenoid molecule. The second strongest peak, ν2, located at 1150 cm^−1^ originates from C–C stretching vibration. The third peak, v3, which is of medium strength located at 1000 cm^−1^ originates from C–CH_3_ in plane rocking vibration. Finally, a weak peak, ν4, located at 958 cm^−1^ originates from combination of C–C stretching, CH_2_ rocking and CH_3_ rocking vibrations. Drastic changes in peak intensities were observed for all bands as the fruit ripens.

Figure 1b represents the outcome of the deconvolution process from the C=C stretching vibration band (ν1). A total of 4 sub bands having distinct peak positions and intensities were observed. This agrees with the findings reported in the configuration and dynamics study of carotenoid components reported by Ruban et al.^41^. The sub bands are located at 1506 (ν1a), 1512 (ν1b), 1518 (ν1c) and 1524 (ν1d) cm^−1^ in average. All of the sub bands originated from the C=C stretching vibration of four different carotenoid molecules. Using the findings from Refs.^18,37,41,46^, we have determined that sub bands ν1a (average peak position at 1506 cm^−1^), ν1b (1512 cm^−1^), ν1c (1518 cm^−1^) and ν1d (1524 cm^−1^) are associated with lycopene, β-carotene, lutein and neoxanthin, respectively. The summary of the molecular assignments for the sub bands are presented in Table 1.

**Table 1:** Molecular assignment for the deconvoluted bands.

Sub bands	Mean peak position (cm^−1^)	Molecular assignment	Target molecule	Pigment color
ν1a	1506	Carotene	Lycopene^45^	Red
ν1b	1512		β-carotene^43^	Orange
ν1c	1518	Xanthophyll	Lutein^46^	Yellow
ν1d	1524		Neoxanthin^41^	Pale yellow^47^

### Feature analysis

In the feature analysis, we decided on four important feature components from the Raman spectra that have the potential to give an accurate assessment of the oil palm fruit ripeness. The features are the position of the peaks, the peak intensity of sub bands, the peak height ratios, and the full width at half maximum (FWHM) of the sub bands.

**Figure 2:**
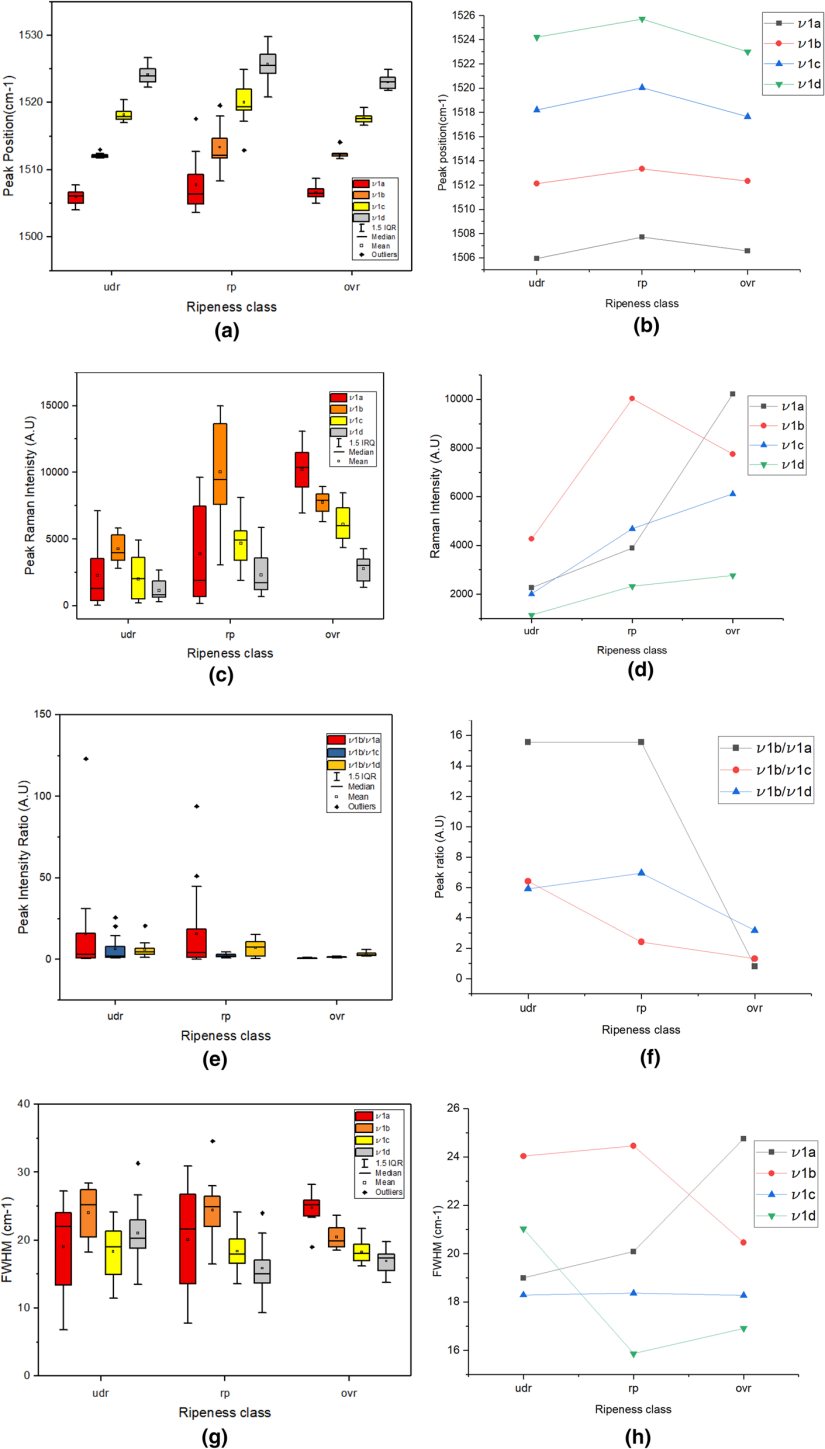
(**a**) Group Box plot of feature 1: Raman peak position. (**b**) Mean plot of feature 1 for 3 levels of ripeness. (**c**) Group Box plot of feature 2: Raman peak intensity. (**d**) Mean plot of feature 2 for 3 levels of ripeness. (**e**) Group Box plot of feature 3: Peak ratio. (**f**) Mean plot of feature 3 for 3 levels of ripeness. (**g**) Group Box plot of feature 4: FWHM. (**h**) Mean plot of feature 4 for 3 levels of ripeness (*udr* under ripe, *rp* ripe, *ovr* over ripe).

The position of the peaks is the characteristic used for discriminating carotenoid molecules. Figure 2a shows a group box plot of extracted peak positions for 3 levels of ripeness. The sub bands ν1a to ν1d has an overall mean peak position of 1506.35, 1512.19, 1518.75 and 1524 cm^−1^ respectively. Sub bands ν1a, ν1b and ν1c starts off with low variance during under ripe state, with the variance peaking during ripe state before reverting back to low variance in over ripe state. On the other hand, the ν1d sub band shows consistently high variance throughout the three states. Figure 2b presents the mean position plot of the sub bands against the 3 ripeness classes. It can be observed that there is no significant change in the position for sub bands ν1a and ν1b throughout the ripening process. However, sub bands ν1c and ν1d show slight drops in position during the transition from ripe to over ripe state.

The second feature, peak intensity of sub bands, represents the concentration of molecules present in the sample. Figure 2c shows a group box plot of extracted peak intensities for the 3 ripeness states. The peak intensities of the sub bands show distinct distributions for each ripeness level. The mean peak intensities of the sub bands are presented in Fig. 2d. During under ripe state, the sub band which corresponds to β-carotene molecule showed the highest mean intensity value, followed, in descending order, by lycopene, lutein and neoxanthin. Subsequently, in ripe state, the mean peak intensity of β-carotene sub band remains highest with an increase of 170% from the previous state. The remaining sub bands corresponding to lycopene, lutein and neoxanthin showed an overall increase in mean intensity values by 51.2%, 126.2% and 35% respectively, with the lutein sub band overtaking the position of the lycopene sub band in the previous order. The descending order of mean peak intensities of the sub bands in the ripe state are as follows: β-carotene, lutein, lycopene, and neoxanthin. Ultimately, in over ripe state, the lycopene sub band attained the highest mean intensity value compared to the rest of the sub bands. The mean intensity of this sub band has increased by 230.5% compared to the previous state. The β-carotene sub band, on the other hand, showed a drop in mean peak intensity by 33.8%, while the rest of the sub bands showed an increasing trend. The mean peak intensities of lutein and neoxanthin sub bands increased by 46% and 83.3%, respectively. The descending order of mean peak intensities in over ripe state are arranged as follows: lycopene, β-carotene, lutein, and neoxanthin.

The third feature, peak height ratio, provides useful information regarding the concentration ratio of a carotenoid pair. By using the peak intensity of band ν1b as a reference, 3 ratios have been produced as shown in Fig. 2e. The ratios are labelled as: ν1b/ν1a, ν1b/ν1c and ν1b/ ν1d. These ratios correlate to the molecule concentration ratios between β-carotene and lycopene, β-carotene and lutein, and finally β-carotene and neoxanthin, respectively. Since each carotenoid molecule possess a unique color in the exocarp, the ratio also correlates to the distribution of exocarp color. The mean peak ratio of the sub bands for 3 ripeness classes are presented in Fig. 2f. During the under ripe state, β-carotene to lycopene peak ratio is observed to be the highest compared to the other ratios. This ratio increases further by 28.64% in ripe state. However, it hits the bottom with a decrease of 96.57% in over ripe state, becoming the lowest among all the other ratios. This indicates that the concentration of lycopene has increased drastically while the concentration of β-carotene has decreased during the transition from ripe to over ripe state. Likewise, the β-carotene to neoxanthin ratio has shown a similar trend. The peak ratio increases by 43.48% from under ripe to ripe state before decreasing later by 64.7% from ripe to over ripe state. Finally, the β-carotene to lutein peak ratio shows a gradually decreasing trend from under ripe to over ripe state.

The last feature, FWHM of Raman bands, provides insights on physical stresses experienced by the molecule. Figure 2g shows a group box plot of extracted FWHM for the 3 ripeness classes. It was observed that the dispersion for all sub bands in under ripe and ripe states are higher compared to over ripe state. Among all the sub bands, the FWHM of lycopene band showed the highest dispersion with standard deviation of 7.1 cm^−1^ during ripe state. Figure 2h presents the mean FWHM plot for all sub bands. During under ripe state, the sub band corresponding to β-carotene has the highest FWHM of all the sub bands. The FWHM of this band continues to increase slightly by 3.49% in ripe state. However, in the subsequent state, it has dropped by 20.3%, becoming the second highest FWHM after the lycopene sub band. The FWHM of the lycopene sub band shows an opposite behavior in contrast to β-carotene sub band. Initially, the FWHM of lycopene sub band dropped by 12.78% during under ripe to ripe state transition. This value subsequently increased by 48.23% from ripe to over ripe state. The last two sub bands corresponding to lutein and neoxanthin shows similar trend with lycopene sub band at a smaller scale.

### Statistical analysis

A total of 15 features from the extracted sub bands were discriminated using statistical evaluation. The ANOVA test for all sub band peak intensities showed positive results. Further post hoc test concluded that β-carotene and lutein sub bands are statistically significant in distinguishing between all states. Therefore, they are potential candidates to be used in the classification analysis. Meanwhile, the peak intensity of neoxanthin sub band is found to be significant only for distinguishing between under ripe and ripe states. The peak intensity of lycopene sub band shows an opposite behavior in contrast to neoxanthin sub band. Hence, the peak intensities of lycopene and neoxanthin were kept as reserve features for classification analysis.

The ANOVA test for peak position of lutein and neoxanthin sub bands showed positive results. A further post hoc test has concluded that the peak position of lutein sub band is significant in discriminating between under ripe and ripe states and between ripe and over ripe states. However, it performs poorly in discriminating between under ripe and over ripe states. The peak position of neoxanthin sub band shows an opposite behavior compared to the lutein sub band. It was found to be statistically significant for distinguishing between ripe and over ripe states. However, it performs poorly in discriminating between under ripe and ripe states, and between over ripe and under ripe states. Nevertheless, the peak positions of lutein and neoxanthin sub bands were kept as reserve features to be used in classification analysis.

The ANOVA test for peak ratio of β-carotene to lutein and β-carotene to neoxanthin sub bands showed positive results. Both features showed similar results in post hoc test. It was observed that both features are useful in distinguishing between ripe and over ripe states. However, they perform poorly in distinguishing between under ripe and ripe states, and between over ripe and under ripe states.

The ANOVA test for FWHM of lutein, β-carotene and neoxanthin showed positive results. A further post hoc test revealed that the FWHM of lycopene and β-carotene sub bands are statistically significant in distinguishing between ripe and over ripe states. However, these features perform poorly in differentiating between under ripe and ripe states. The FWHM of neoxanthin sub band is statistically significant in distinguishing between under ripe and ripe states and between ripe and over ripe states. However, it performs poorly when differentiating between under ripe and over ripe states. Therefore, the FWHM of lycopene, β-carotene and neoxanthin are kept as reserved features.

### Classification analysis

The statistical analysis conducted earlier helps to reduce the 15 features into 9 features. The selected features are separated into two classes, namely: main features and reserved features. Main features are features that show statistical significance across all classes of ripeness in post hoc test whereas reserved features are features that are statistically significant for at most two ripeness classes. The results for classification analysis are tabulated in Table 2. The first classification analysis conducted using only two predictors: peak intensities of β-carotene and lutein sub bands, showed a maximum accuracy of 80.4 % using the SVM fine Gaussian kernel classifier. In further analysis using four predictors: peak intensities of lycopene, β-carotene, lutein and neoxanthin sub bands, a maximum accuracy of 91.3% has been obtained using SVM medium Gaussian kernel classifier and weighted KNN classifier, which is an increase of 10.9% in prediction accuracy compared to the previous analysis with only two features. In the final analysis, six predictors: peak intensities of lycopene, β-carotene, lutein and neoxanthin sub bands, and peak positions of β-carotene and lutein were employed. A maximum accuracy of 100% has been obtained using KNN classifier with fine KNN, which is the highest prediction accuracy observed compared to all other classifiers. The next most accurate prediction is shown by 3 classifiers: quadratic discriminant, SVM cubic kernel and SVM medium Gaussian kernel, with an accuracy of 97.8%. Finally, an accuracy of 95.7% was observed for one classifier: SVM with quadratic kernel.

**Table 2:** Summary of classification analysis results.

Classifier type	Predictor variable	Accuracy (%)
Fine KNN	4 Peak intensity (lycopene, β-carotene and lutein and neoxanthin) and 2 peak position (lutein and neoxanthin)	100
SVM cubic kernel		97.8
SVM medium Gaussian		
Quadratic discriminant		
SVM quadratic kernel		95.7
Weighted KNN	4 Peak intensity (lycopene, β-carotene and lutein and neoxanthin)	91.3
SVM medium Gaussian kernel		
SVM fine Gaussian kernel	2 Peak intensity (β-carotene and lutein)	80.4

## Discussion

**Figure 3:**
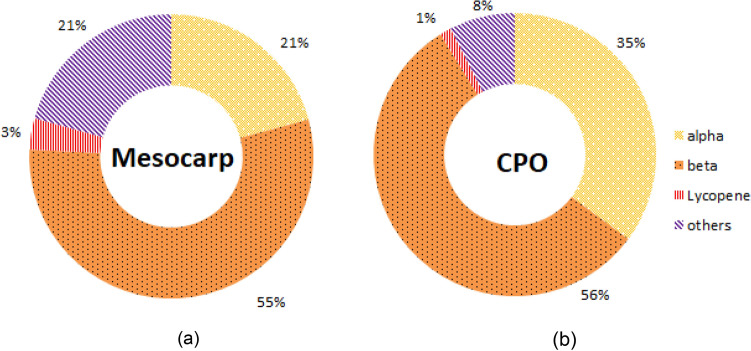
Carotenoid composition (%) of ripe fruit Tenera variety (**a**) Mesocarp extract^48^, (**b**) Crude palm oil^50^.

**Figure 4:**
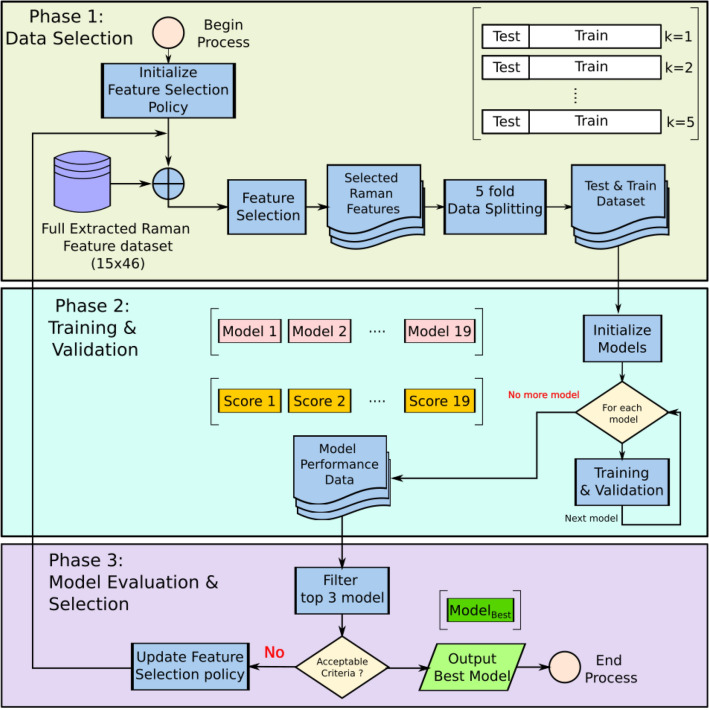
Classification analysis method.

Previously, several works have reported the carotenoid composition in oil palm mesocarp and crude palm oil (CPO). As illustrated in Fig. 3 in the Appendix, β-carotene accounts for 55% in the mesocarp and 56% in the CPO respectively. Besides that, lycopene accounts for 3% in the mesocarp and 1% in the CPO. However, to our best knowledge there has been no information on the carotenoid composition in the oil palm exocarp that has been reported. It was observed that the mean peak intensity of ν1b sub band corresponding to β-carotene molecule was the highest among the other sub bands during under ripe and ripe states. This agrees with findings of previous researches^48–50^ which claim that β-carotene is the highest constituent among the total carotenoids found in oil palm fruits. The increase in β-carotene peak intensity indicates that the fruit is progressing towards maturity. Since β-carotene is an orange-colored pigment, the intensity of orange pigment increases as the fruit undergoes maturity. However, we observed that there is a decrease in β-carotene concentration throughout the ripe to over ripe state which has not been previously reported. The concentration of lycopene observed in this study increases gradually throughout the ripening process. It was found to be at its maximum during over ripe state. This indicates that the concentration of lycopene kept increasing even after the fruitlets have attained maturity. Since lycopene is a red-colored pigment, the increase in lycopene concentration results in the increase of red color in the fruitlets. The redness of over ripe oil palm is expected to be higher compared to ripe classes. This result agrees with previous findings of Patkar et al. whereby the highest red value in RGB is found in over ripe fruitlets^51^. A similar observation was reported by Trebolazabala et al. for lycopene content observed in the Raman spectra of red tomatoes^36,37^. The concentration of lutein and neoxanthin molecules increase steadily throughout the ripening process. Since lutein appears yellow-colored in nature, the appearance of yellow color in the skin also increases gradually. Chong et al. has reported that ripe fruits appear to be more yellowish than reddish in color^52^. This report on the yellow color in ripe fruits correlates with the increment in lutein concentration of ripe fruits observed in this study.

A summary of 19 oil palm ripeness classifiers from previous literature along with their predictor variables and prediction accuracies are tabulated in Appendix Table 3. Only 10 out 19 classifiers reported in previous studies have achieved a prediction accuracy of more than 90%. Furthermore, only 4 out of 19 classifiers reported in the literature have achieved a prediction accuracy greater than 95%. Thus far, only one previous study has reported a prediction accuracy of 100%, using spectral reflectance features obtained from hyperspectral camera^32,34^. However, hyperspectral cameras are computationally expensive due to its requirement of high storage capacity for saving and retrieving the information. Hence, the best benchmark for oil palm ripeness classification accuracy reported thus far is 98.9% using SVM classifier which relies on visible light imaging. Table 2 previously has shown the top 8 classifiers trained in this study with fine KNN classifier achieving a maximum prediction accuracy of 100%. This classifier has outperformed all the other classifiers previously reported in the literature as listed in Appendix Table 3.

## Conclusion and future perspective

This study demonstrates that the Raman spectroscopy is a rapid, non-invasive and reliable technique for assessing the ripeness of oil palm fruits. Furthermore, this technique is robust against the effects of lighting and noise caused by moisture. The analysis has shown that β-carotene, lycopene, lutein and neoxanthin are the carotenoids found in the oil palm fruit skin. The band originating from C=C double bond stretching vibration (ν1) contains an abundance of information to assess the ripeness state of the oil palm fruitlets. A total of 15 features were extracted and identified from this band through a deconvolution process with 9 features statistically tested to be significant: 4 peak intensities (ν1a–d), 2 peak positions (ν1c,d) and 3 FWHM (ν1a,b,d). From these, 6 features were chosen to achieve a classification accuracy of 100% using fine KNN classifier with peak intensities ν1a–d and peak positions ν1c,d as predictors.

The scope of the existing work is limited to the investigation of the relationship between the oil palm fruit ripeness level and the Raman spectra of carotenoid content obtained from the exocarp. In the future, the existing work can be extended to include the correlation study between oil content and free fatty acid (FFA) levels. Correlating carotenoid content from Raman spectra with oil content and FFA levels will aid the establishment of quantitative prediction models. Hence the prospective users will benefit by obtaining multiple information simultaneously (ripeness, oil content, and FFA level) from a single scan. The deconvolution process employed in existing work was aided by human interaction especially during initialization of parameters. Due to heavy overlapping of carotenoid bands in narrow bandwidth, traditional automated peak finding algorithms fail to operate efficiently. Hence, further investigations into automated deconvolution algorithms are required to bridge this gap. Although the existing study utilizes benchtop Raman spectrometer, similar results are expected to be obtained using a portable device. The proposed method has the potential to be a rapid and in-situ assessment tool for ripeness classification in the oil palm industry.

## Appendix

See Figs. 3, 4 and Table 3.

**Table 3:** Summary of classifiers reported in the literature.

Classifier type	Detection technique	Predictors	Accuracy (%)	Ref.
CRT	Fluorescence sensor	Blue-to-red fluorescence ratio	89.7	^30^
SGBT		Flavonoids and anthocyanins content	87.7	^53^
LD	NIR spectrometer	5 PCA variable	81.7	^29^
QD	Multispectral sensor	8 PCA variable	86.7	^31^
ED	Hyper spectral camera	Average reflectance	100	^34^
ANN-MLP		Spectral bands between 750 to 900 nm	94.54	^32^
KNN		ROI based color histogram	94	^54^
ANN-MLP	Imaging	R, G, B, r, g, b, H, S, I, RI	93.5	^21^
ANN		ROI based color histogram	91.3	^25^
GA		A, B and Intensity (CIE value)	67.1	^24^
K-means clustering		R, G, B, r, g, b, H, S, I, RI	93.5	^22^
ANN		HSI	70.0	^18^
Kullback–Leibler distance		Relative entropy	96	^17^
Fuzzy logic		Mean RGB value	86.67	^20^
Neuro-fuzzy system		RGB value	73.3	^19^
CNN		Color moments	92.0	^23^
KNN		Multiband spectral reflectance	80.7	^35^
SVM		Color moment	96.59	^5^
SVM		YCbCr or YUV	98.9	^26^
